# Occurrence of cefotaxime-resistant and beta-lactamase-producing Enterobacterales in poultry from small-scale farms in the Copperbelt province of Zambia

**DOI:** 10.1093/jacamr/dlaf125

**Published:** 2025-07-22

**Authors:** Situmbeko J Nasilele, Misheck Shawa, Harvey K Kamboyi, Bruno S J Phiri, Herman Chambaro, Tapiwa Lundu, Mike Nundwe, Angela Lungu, Ladslav Moonga, Masahiro Kajihara, Hirofumi Sawa, Yasuhiko Suzuki, Hideaki Higashi, Amon Siame, Ntombi B Mudenda, Mudenda B Hang’ombe, Kaampwe Muzandu

**Affiliations:** Biomedical Sciences Department, School of Veterinary Medicine, University of Zambia, Lusaka 10101, Zambia; Hokudai Center for Zoonosis Control in Zambia, University of Zambia, Lusaka 10101, Zambia; Division of Infection and Immunity, International Institute for Zoonosis Control, Hokkaido University, N20 W10, Kita-ku, Sapporo 001-0020, Japan; Department of Para-Clinical Studies, School of Veterinary Medicine, University of Zambia, Lusaka 10101, Zambia; Ministry of Fisheries and Livestock, Central Veterinary Research Institute, Lusaka 10101, Zambia; Biomedical Sciences Department, School of Veterinary Medicine, University of Zambia, Lusaka 10101, Zambia; Institute of Basic and Biomedical Sciences, Levy Mwanawasa Medical University, Lusaka 10101, Zambia; Department of Disease Control, School of Veterinary Medicine, University of Zambia, Lusaka 10101, Zambia; Division of Infection and Immunity, International Institute for Zoonosis Control, Hokkaido University, N20 W10, Kita-ku, Sapporo 001-0020, Japan; Hokudai Center for Zoonosis Control in Zambia, University of Zambia, Lusaka 10101, Zambia; Division of International Research Promotion, International Institute for Zoonosis Control, Hokkaido University, N20 W10, Kita-ku, Sapporo 001-0020, Japan; Hokudai Center for Zoonosis Control in Zambia, University of Zambia, Lusaka 10101, Zambia; Institute for Vaccine Research and Development (HU-IVReD), Hokkaido University, N21 W11, Kita-ku, Sapporo 001-0020, Japan; Division of Bioresources, International Institute for Zoonosis Control, Hokkaido University, Kita-ku, Sapporo, Japan; Division of Infection and Immunity, International Institute for Zoonosis Control, Hokkaido University, N20 W10, Kita-ku, Sapporo 001-0020, Japan; Department of Pathology and Microbiology, School of Medicine, University of Zambia, Lusaka 10101, Zambia; Department of Clinical Studies, School of Veterinary Medicine, University of Zambia, Lusaka 10101, Zambia; Division of Infection and Immunity, International Institute for Zoonosis Control, Hokkaido University, N20 W10, Kita-ku, Sapporo 001-0020, Japan; Division of Research and Innovation, Copperbelt University, Jambo Drive, Riverside, Kitwe 10101, Zambia; Biomedical Sciences Department, School of Veterinary Medicine, University of Zambia, Lusaka 10101, Zambia

## Abstract

**Background:**

The inappropriate use of antimicrobials in poultry farming is associated with the emergence of antimicrobial-resistant Enterobacterales. This cross-sectional study aimed to identify various cefotaxime-resistant and β-lactamase-producing Enterobacterales and characterize their antimicrobial resistance profiles.

**Methods:**

Pooled cloacal and meat samples collected from market-ready broiler chickens in Kitwe and Ndola districts of Zambia were screened for cefotaxime-resistant Enterobacterales. The samples were inoculated on MacConkey agar supplemented with 1 mg/L cefotaxime. The cefotaxime-resistant isolates were further subjected to antimicrobial susceptibility tests. Further, the isolated cefotaxime-resistant Enterobacterales were analysed for *bla*_CTX-M_, *bla*_TEM_, *bla*_OXA-1-like_ and *bla*_SHV_ genes using PCR and Sanger sequencing.

**Results:**

From a total of 114 pooled samples, 81 (71.1%) cefotaxime-resistant Gram-negative strains were isolated. These were dominated by *Escherichia coli* (77.8%) followed by *Klebsiella pneumoniae* (6.2%), *Pseudomonas* spp. (6.2%), *Acinetobacter baumannii* (4.9%), *Pseudomonas aeruginosa* (2.5%), *Enterobacter* spp. (1.2%) and *Comamonas aquatica* (1.2%). Furthermore, 64.2% of the 81 isolates exhibited multidrug resistance with high resistance (>64%) to ampicillin, co-trimoxazole and tetracycline. The results also showed that 66.7% of the isolates harboured at least one of the four tested *bla* genes (*bla*_CTX-M_, *bla*_TEM_, *bla*_OXA-1-like_ and *bla*_SHV_), with the commonest being *bla*_CTX-M_ (58%) and *bla*_TEM_ (45.7%).

**Conclusions:**

The study revealed a high prevalence of cefotaxime-resistant Enterobacterales and multidrug resistance involving medically important antibiotics. Four *bla* genes (*bla*_CTX-M_, *bla*_TEM_, *bla*_OXA-1-like_ and *bla*_SHV_) were identified. Our results highlight the need to strengthen antimicrobial stewardship programmes and optimize antimicrobial use in poultry farming.

## Introduction

Zambia’s poultry industry is a significant part of the agricultural sector, providing a substantial source of protein and employment for the local population.^1^ Particularly, broiler chicken production is a continuously growing sector due to high demand over the past few years. Broilers are usually raised in intensive farming systems, where large numbers of birds are kept in confined spaces, emphasizing rapid growth and high turnover.^2^ These conditions present the harsh challenge of disease management as poultry requires strict biosecurity to prevent the introduction and spread of pathogens.^3^ According to the 2023 Livestock Survey Report, the major problem encountered by broiler chicken-raising households in Zambia at 41.6% was disease.^4^ This, coupled with increased consumer demand, compels farmers to resort to antibiotic use for prophylaxis and growth promotion. Thus, the growth of the poultry industry in Zambia has led to the concomitant increase in antimicrobial use (AMU) with the most sold antibiotics being penicillins, tetracyclines and sulfonamides.^5,6^

Several reports globally have collectively highlighted the significant role of AMU in chicken farming in the development and spread of antimicrobial resistance (AMR), emphasizing the need for responsible antimicrobial practices to mitigate this global health threat.^7–11^ Other than misuse and overuse of antimicrobials in the poultry production, AMR also spreads via contamination with AMR pathogens from the environment along the food chain to a larger extent.^2,12–14^ Contamination of chicken meat occurs from various sources throughout the production (feed and water contamination) and processing (gut microbiota) stages. Contaminated feed or water introduces AMR bacteria that can colonize the intestines and eventually, the meat during processing if handling is poor. Muscles are sterile in healthy chickens, however, poor handling during processing may introduce bacteria from the digestive tract, skin and feathers.^15,16^

The gut microbiome in chickens consists of a diverse community of microorganisms but most of isolates that have been associated with AMR are Enterobacterales, especially *Escherichia coli* and *Salmonella* spp.^17,18^ These isolates have been resistant to critically important antibiotics such as third-generation cephalosporins (3GCs), fluoroquinolones and carbapenems.^19^ Resistance to 3GCs is usually due to β-lactamases, particularly extended-spectrum β-lactamases (ESBLs).^20^ The β-lactamase genes are often located on plasmids that also carry genes conferring resistance to other classes of antibiotics, such as aminoglycosides, fluoroquinolones or tetracyclines.^21^ This indicates that the use of one antibiotic can co-select for resistance to multiple antibiotics, complicating treatment options and leading to multidrug resistance (MDR).^22^ In clinical cases involving MDR pathogens, carbapenems are the favoured last resort drugs for treatment. However, carbapenem resistance has also become an urgent global health threat due to the limited treatment options available for infections caused by carbapenem-resistant organisms.^23^ Carbapenems are a class of beta-lactam antibiotics known for their broad-spectrum activity and strong resistance to most beta-lactamase enzymes produced by bacteria. Carbapenem resistance is a growing public health concern in Zambia both in human and animal health. A study at a large tertiary referral hospital in Zambia reported carbapenem resistance in *Pseudomonas aeruginosa* and *Acinetobacter* species to be 6% and 18.2%, respectively.^24^ In poultry, a study conducted on commercial and medium-/small-scale farms in Zambia reported a 5.7% ESBL-*E. coli* resistance to imipenem.^25^

Despite the increasing reports on AMR among poultry isolates in Zambia,^25,26^ many studies focus on the phenotypic characterization of *E. coli* or *Salmonella* spp.^27,28^ However, the reservoirs of AMR genes are broad and include various Enterobacterale species. This study aimed to identify poultry associated cefotaxime-resistant and β-lactamase-producing Enterobacterales. Furthermore, the study also aimed to characterize Enterobacterale isolates for their antimicrobial resistance patterns and AMR genes.

## Materials and methods

### Study area and sampling

The study was conducted in the Copperbelt province of Zambia, which has a total human population of 2 757  539^29^ possessing the highest number of households raising broiler chickens at 12 521/46 927 (26.7%).^4^ As part of the national AMU and AMR surveillance project, pooled cloacal (*n* *=* 57) and meat samples (*n* *=* 57) were collected between April and September 2024 from 57 randomly selected small-scale broiler farms in Ndola (*n* *=* 26) and Kitwe (*n* *=* 31) districts. One cloacal and one meat swab were collected independently per 100 birds on each farm. Only small-scale farms with 100–1000 birds at point of selling were selected in this study, thus farms with more than 100 birds had multiple cloacal and meat swabs collected. All the birds were slaughtered by the research team under carefully monitored field conditions following strict hygiene measures to reduce external contamination. The swabs were then collected aseptically immediately post-slaughter and after defeathering. The swabs were preserved in Amies transport media (Mantacc, Miraclean Technology Co., Ltd, China) and chilled on ice during transportation to the laboratory at the University of Zambia, School of Veterinary Medicine for processing and analysis. The cloacal swabs from multiple chickens on a single farm were pooled as one sample during laboratory processing and the same was done for all meat swabs collected.

### Isolation and identification of cefotaxime-resistant Enterobacterales

The separately pooled cloacal and meat swabs were pre-enriched in Buffered Peptone Water (BPW) (HiMedia, Pvt. Ltd, India) by transferring the swabs to 9 mL of BPW and incubating at 37°C for 18 h. Ten microliters of the overnight cultures was inoculated on MacConkey agar (HiMedia, Pvt. Ltd, India) supplemented with 1 mg/L of cefotaxime and incubated at 37°C for 18 h. The growth observed was regarded as presumptive β-lactamase-producing Enterobacterales.^30^

### Molecular characterization of cefotaxime-resistant Enterobacterales

Molecular characterization of cefotaxime resistance was conducted by screening for the common β-lactamase-encoding (*bla*) genes (*bla*_CTX-M_, *bla*_TEM_, *bla*_OXA-1-like_ and *bla*_SHV_) using validated PCR primers shown in Table 1.^31–35^ Genomic DNA was extracted from 3GC-resistant isolates using DNAzol^®^ Genomic DNA Isolation Reagent (Molecular Research Centre, Inc., USA) according to the manufacturer’s instructions. PCR was performed using TaKaRa ExTaq HS version (TaKaRa, Japan), which consists of PCR buffer, dNTPs and Ex Taq DNA polymerase separately. The PCR volume was 25 μL and the conditions for *bla*_CTX-M_, *bla*_TEM_ and *bla*_OXA-1-like_ were initial denaturation at 94°C for 1 min, followed by 30 cycles of template denaturation at 94°C for 30 s, primer annealing at 58°C for 30 s and extension at 72°C for 30 s, then a final extension at 72°C for 5 min. The PCR conditions for *bla*_SHV_ were the same except for the annealing temperature, which was 56°C for 30 s. PCR products were visualized under UV light after electrophoresis using 1.5% agarose gel. Pairwise comparisons for proportions in R was used to identify statistically significant differences between proportions of *bla* genes between Ndola and Kitwe.

**Table 1.** Specific primers used in this study

Primer name	Sequence (5′–3′)	Product size (bp)	Reference
CTX-MA1	^a^SCSATGTGCAG^b^YACCAGTAA	544	^31^
CTX-MA2	CCGC^c^RATATGRTTGGTGGT		
TEM-F	GTATCCGCTCATGAGACAATA	717	^32^
TEM-R	AGAAGTGGTCCTGCAACTTT		
OXA-1-F	GGCACCAGATTCAACTTTCAAG	564	^33^
OXA-1-R	GACCCCAAGTTTCCTGTAAGTG		
SHV-F	AGGATTGACTGCCTTTTTG	392	^34^
SHV-R	ATTTGCTGATTTCGCTCG		
yaiO-F	TGATTTCCGTGCGTCTGAATG	115	^35^
yaiO-R	ATGCTGCCGTAGCGTGTTTC		

^a^S = G or C.

^b^Y = C or T.

^c^R = A or T.

### Identification of cefotaxime-resistant isolates

Isolated cefotaxime-resistant isolates were first subjected to PCR to detect the *E. coli*-specific *yaiO*. Briefly, PCR products were purified using the Promega Wizard^®^ SV Gel and PCR Clean-Up System purification kit (Madison, WI, USA), according to the manufacturer’s instructions. Further, all cefotaxime-resistant isolates that were negative for *yaiO* were identified through 16S rRNA Sanger sequencing using BigDye Terminator v.3.1 (Applied Biosystems, Waltham, MA, USA). The obtained sequences were processed and assembled into consensus sequences using SnapGene v.7.2. The assembled 16S rRNA encoding consensus sequences were blasted in National Center for Biotechnology Information using BLAST search optimized for highly similar sequences (megablast) for species identification.

### Antimicrobial susceptibility testing (AST)

The AST was performed using the disc diffusion method described by Kirby and Bauer^36^ on Mueller–Hinton (MH) agar (Oxoid, Germany). Sterile saline was used to emulsify the colonies to achieve turbidity equivalent to 0.5 McFarland standard, which is equivalent to 1.5 × 10^8^ cfu/ml.^36^ Suspensions were spread onto MH agar, placed the antibiotic discs and incubated at 37°C for 18 h. The antibiotic discs used were ampicillin (10 μg), sulfamethoxazole/trimethoprim (25 μg), ciprofloxacin (5 μg), tetracycline (30 μg), gentamicin (10 μg), chloramphenicol (30 μg), azithromycin (15 μg) and imipenem (10 μg). The interpretation was based on the guidelines in the Clinical and Laboratory Standards Institute guidelines.^37^ The *E. coli* strain ATCC 25922 was used as a positive control. These antibiotics were selected based on prescribing patterns in human and veterinary medicine in Zambia where they are among those highly used for disease prevention and treatment, and in small-scale poultry farming.^6,7,25,38,39^

A dendrogram was created using AST data and *bla* genes to group isolates according to their AMR profiles and presence/absence of *bla* genes. The dendrogram was constructed using R version 4.4.1. To prepare and organize the data, the tidyverse, reshape2 and dplyr packages were used. Circlize, igraph and ComplexHeatmap packages were then used for hierarchical clustering and construction of the dendrogram. This produced the cluster-based visualizations of AMR patterns.

### Phenotypic ESBL confirmation

Phenotypic confirmation of ESBL was conducted on 40 randomly selected isolates using the combination disc test. To this end, a MH agar plate was inoculated with a 0.5 MacFarland suspension of the isolate to be tested. Next, cefpodoxime 10 µg and cefpodoxime/clavulanic acid 10/10 µg discs were placed 25 mm (centre-to-centre) and the plates incubated for 18 h at 37°C. An ESBL-positive result was recorded when the zone diameter of the disc with cefpodoxime/clavulanic was at least 5 mm (≥5 mm). The standard ESBL control strain *Klebsiella pneumoniae* ATCC 700603 was used as positive control while *E. coli* ATCC 25922 was used as negative control.

### Data analysis

Data were imported in R v.4.4.1 and manipulated using dplyr^40^ before visualization with ggplot2^41^ and ComplexHeatmap.^42^ Categorical data were analysed by the Chi-square test using the epiR package.^43^

### Ethical approval

Ethical approval was sought from the Excellence in Research Ethics and Science Converge, Lusaka, Zambia (reference number 2023-Apr-001). Before specimen collection, informed consent was sought from farm owners to collect chicken samples from their farms.

## Results

### Prevalence of cefotaxime-resistant Enterobacterales

A total of 57 pooled cloacal and 57 pooled chicken meat swabs from 57 small-scale broiler farms in Ndola (*n* *=* 26) and Kitwe (*n* *=* 31) were screened for cefotaxime resistance. Of the 114 pooled samples (57 from cloaca and 57 from meat), only 81 were cefotaxime-resistant. Out of these 81 cefotaxime-resistant isolates, 51.9% (42/81) were from the cloaca while 48.1% (39/81) were from meat (Table S1, available as Supplementary data at *JAC-AMR* Online). From the cloaca, only *E. coli* (42/81, 51.9%) was isolated while the 39/81 (48.1%) isolates from meat were composed of *E. coli* (21/39, 53.8%), *K. pneumoniae* (5/39, 12.8%), *Pseudomonas* spp. (5/39, 12.8%), *A. baumannii* (4/39, 10.3%), *P. aeruginosa* (2/39, 5.1%), *Enterobacter* spp. (1/39, 2.6%) and *Comamonas aquatica* (1/39, 2.6%) (Table 2).

**Table 2.** Prevalence of MDR species (*n* = 81)

Species	Cefotaxime resistance per source		Overall cefotaxime resistance *n* (%)	MDR rate	
	Cloacal swabs	Meat swabs		*n*	%
*E. coli*	42	21	63 (77.8)	46	73
*K. pneumoniae*	0	5	5 (6.2)	4	80
*Pseudomonas* spp.	0	5	5 (6.2)	2	40
*A. baumannii*	0	4	4 (4.9)	0	0
*P. aeruginosa*	0	2	2 (2.5)	0	0
*Enterobacter* spp.	0	1	1 (1.2)	0	0
*C. aquatica*	0	1	1 (1.2)	1	100

Forty-six of the 81 cefotaxime-resistant isolates were from Ndola district, of which 25/46 (54.3%) were from the cloaca and 21/46 (45.7%) were from the meat. From Kitwe district, a total of 35 isolates were cefotaxime-resistant of which 17/35 (48.6%) were from the cloaca while 18/35 (51.4%) were from the meat. In summary, of all the 81, cefotaxime-resistant isolates in this study, 42/81 (51.9%) were from the cloaca while 39/81 (48.1%) were from the meat. The prevalence of 3GC resistance was 70.1% (81/114).

**Figure 1.**
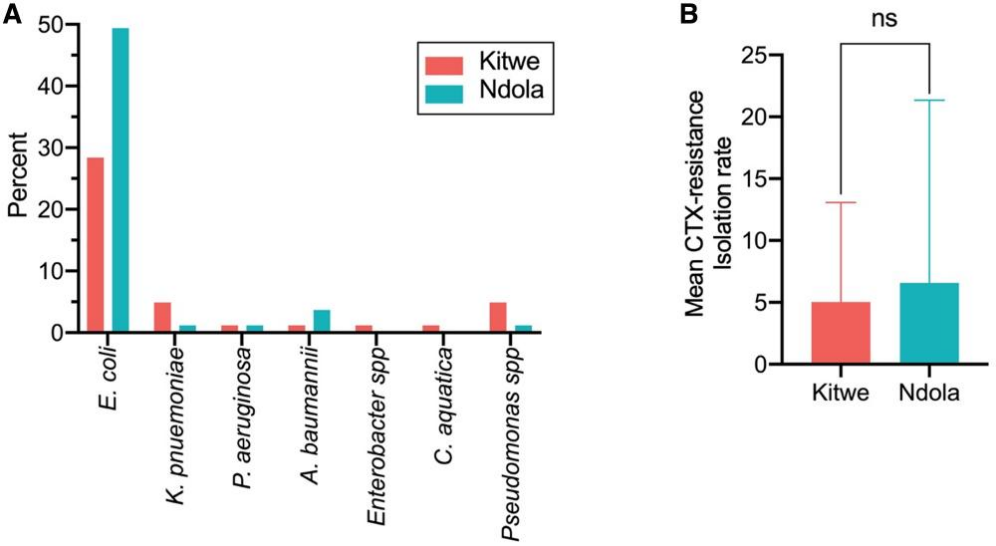
Distribution of cefotaxime (CTX)-resistant Enterobacterales from poultry by district. (a) The proportion cefotaxime-resistant *E. coli* was significantly higher in both districts compared to other species isolated. (b) On average (mean ± SD), there was no significant difference in the overall isolation rate of cefotaxime-resistant Enterobacterales from poultry in Kitwe and Ndola. Statistical significance was calculated using a paired two-tailed *t*-test, *P* = 0.5753.

The bacterial species identified were *E. coli* (63/81, 77.8%), *Klebsiella pneumoniae* (5/81, 6.2%), *Pseudomonas* spp. (5/81, 6.2%), *Acinetobacter baumannii* (4/81, 4.9%), *Pseudomonas aeruginosa* (2/81, 2.5%), *Enterobacter* spp. (1/81, 1.2%) and *Comamonas aquatica* (1/81, 1.2%). The *Pseudomonas* spp. and *P. aeruginosa* were separated due to the blast search not identifying the *Pseudomonas* spp. to species level. The proportion of cefotaxime-resistant *E. coli* was significantly higher in both districts compared to other species isolated, (Figure 1a). Additionally, there was no significant difference in the overall isolation rate of cefotaxime-resistant Enterobacterales from poultry in Kitwe and Ndola, *P* = 0.5753 (Figure 1b). Furthermore, there was no statistical difference in the prevalence of *E. coli* in the groups of chicken population sizes from where the samples were collected, *P* > 0.05 (Table S2).

### *Distribution of* bla *genes among cefotaxime-resistant isolates*

**Figure 2.**
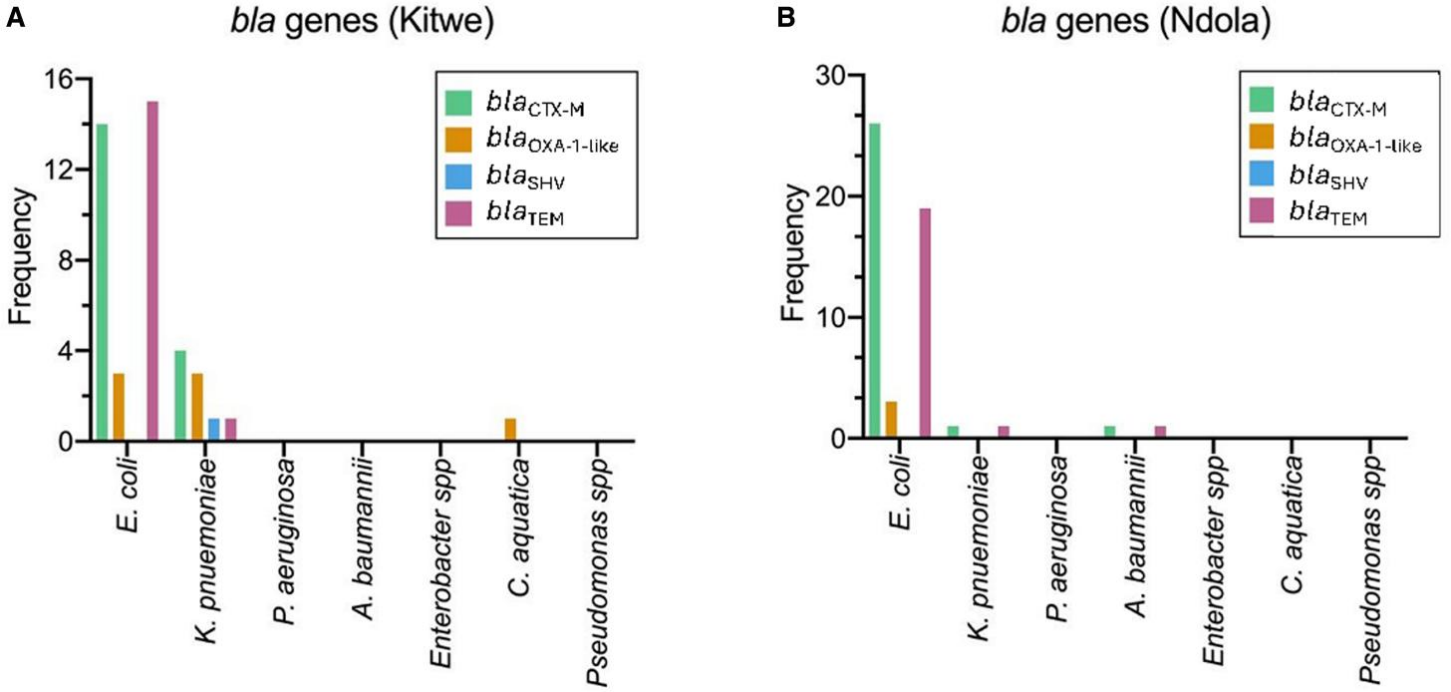
Frequency of *bla* genes among the cefotaxime-resistant isolates in Kitwe and Ndola. Target genes by district. *Bla* genes were commonly detected among *E. coli* isolates from both districts, especially *bla*_CTX_M_ and *bla*_TEM_. At least one *bla* gene was detected in *K. pneumoniae*, *A. baumannii* (Ndola) and *C. aquatica* (Kitwe) in contrast to *P. aeruginosa* and other *Pseudomonas* species in which none were detected.

Characterization of cefotaxime-resistant isolates was carried out by screening for *bla* genes (*bla*_CTX-M_, *bla*_TEM_, *bla*_SHV_ and *bla*_OXA-1-like_). The results showed that 66.7% (54/81) of the isolates harboured at least one *bla* gene (Figure 2). This was dominated by the *bla*_CTX-M_ gene (47/81, 58%), followed by *bla*_TEM_ (37/81, 45.7%), *bla*_OXA-1-like_ (10/81, 12.3%) and *bla*_SHV_ (1/81, 1.2%). There was no statistical difference in the proportions of *bla* genes between Ndola (*bla*_CTX-M_ = 29/46, *bla*_TEM_ = 21/46, *bla*_OXA-1-like_ = 3/46 and *bla*_SHV_ = 0/46) and Kitwe (*bla*_CTX-M_ = 18/35, *bla*_TEM_ = 13/35, and *bla*_OXA-1-like_ = 7/35 and *bla*_SHV_ = 1/35) at 95% CL (*bla*_CTX-M_, *P* = 0.41; *bla*_TEM_, *P* = 0.71; *bla*_OXA-1-like_, *P* = 0.14; *bla*_SHV_, *P* = 0.44) (Figure 2). On bacterial species level, 77.8% (49/63) of the *E. coli* isolates were positive for at least one *bla* gene, of which 44.4% harboured *bla*_CTX-M_ and *bla*_TEM_ in co-existence. Each of the five isolates of *K. pneumoniae* tested positive for at least one of the tested *bla* genes (*bla*_CTX-M_, *bla*_TEM_, *bla*_OXA-1-like_ and *bla*_SHV_), while 1/4 *A. baumannii* was positive for *bla*_CTX-M_ and *bla*_TEM_ gene and 1/1 *C. aquatica* possessed the *bla*_OXA-1-like_ gene (Figure 2). Contrarily, *P. aeruginosa*, *Pseudomonas* spp. and *Enterobacter* spp. were negative for the tested *bla* genes.

**Figure 3.**
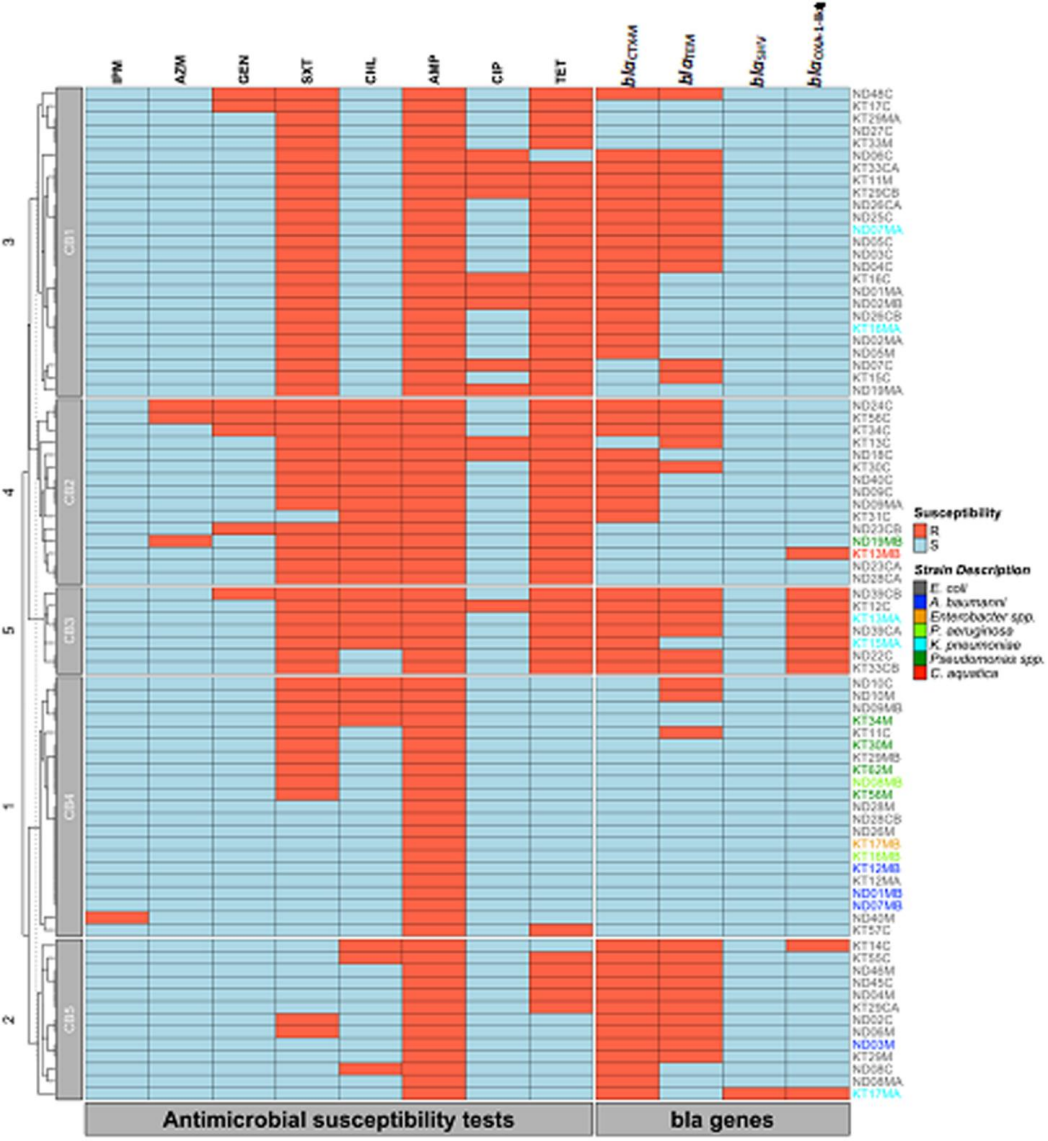
Heatmap showing antimicrobial susceptibility profiles and β-lactamase-encoding genes. IPM, imipenem; AZM, azithromycin; GEN, gentamicin; SXT, sulfamethoxazole and trimethoprim; CHL, chloramphenicol; AMP, ampicillin; CIP, ciprofloxacin and TET, tetracycline. For the antimicrobial susceptibility tests: red rectangles indicate resistance, blue: susceptible. For the *bla* genes, the red and blue rectangles indicate positive (presence) and negative (absence), respectively.

A dendrogram utilizing AST results and *bla* genes showed that, the *Pseudomonas* spp., *P. aeruginosa* and *A. baumannii* clustered together in CB4, which was generally characterized by the absence of *bla* genes (Figure 3). Furthermore, most isolates in this cluster did not exhibit MDR.

### Phenotypic confirmation of ESBL production

Of the 40 randomly selected isolates on which the combination disc test was performed, 80% (32/40) were confirmed to be ESBL producers. *E. coli* was dominating at 84.4% (27/32) and all the five *K. pneumoniae* isolates in this study were found to be ESBL producers (Table S3).

### High MDR rates among the Enterobacterales

All 81 strains isolated from cloacal and meat swabs were subjected to antimicrobial susceptibility testing (AST) against eight classes of antibiotics (Table 3). The results showed the highest resistance to ampicillin (81/81, 100%), followed by co-trimoxazole (58/81, 71.6%), tetracycline (52/81, 64.2%), chloramphenicol (27/81, 33.3%), ciprofloxacin (12/81, 14.8%), gentamicin (7/81,8.6%), azithromycin (3/81, 3.7%) and imipenem (1/81, 1.2%) (Figure 3). Isolates categorized as ‘intermediate’ (I) were interpreted as ‘susceptible, increased exposure’ according to European Committee on Antimicrobial Susceptibility Testing (EUCAST) guidelines^44^ and were considered ‘susceptible’ in this study.

**Table 3.** AST according to bacterial species

Antibiotics	AMP		TET		SXT		CHL		CIP		GEN		AZM		IPM	
Species	S^a^	R^b^	S	R	S	R	S	R	S	R	S	R	S	R	S	R
*E. coli* (*n* = 63)	0	63	19	44	16	47	41	22	51	12	56	7	61	2	62	1
*K. pneumoniae* (*n* = 5)	0	5	1	4	1	4	3	2	5	0	5	0	5	0	5	0
*Pseudomonas* spp. (*n* = 5)	0	5	4	1	0	5	3	2	5	0	5	0	4	1	5	0
*A. baumannii* (*n* = 4)	0	4	2	2	4	0	4	0	4	0	4	0	4	0	4	0
*P. aeruginosa* (*n* = 2)	0	2	2	0	1	1	2	0	2	0	2	0	2	0	2	0
*Enterobacter* spp. (*n* = 1)	0	1	1	0	1	0	1	0	1	0	1	0	1	0	1	0
*C. aquatica* (*n* = 1)	0	1	0	1	0	1	0	1	1	0	1	0	1	0	1	0
Totals (*n* = 81)	0	81	29	52	23	58	54	27	69	12	74	7	78	3	80	1

AMP, ampicillin; TET, tetracycline; SXT, sulfamethoxazole and trimethoprim; CHL, chloramphenicol; CIP, ciprofloxacin; GEN, gentamicin; AZM, azithromycin and IPM, imipenem.

^a^Susceptible.

^b^Resistant.

Additionally, the susceptibility profiles of strains showed a high prevalence of MDR 64.2% (52/81), being resistant to at least three antibiotic classes. Specifically, MDR was detected in *K. pneumoniae* (4/5, 80%), *E. coli* (46/63, 73%), *Pseudomonas* spp. (2/5, 40%) and *C. aquatica* (1/1, 100%), while *P. aeruginosa*, *A. baumannii* and *Enterobacter* species did not exhibit MDR (Table 2).

## Discussion

Our study findings highlight a high prevalence of cefotaxime-resistant and ESBL-producing Enterobacterales in broiler farms within Ndola and Kitwe districts of Zambia. The overall cefotaxime resistance rate of 70.1% among cloacal and meat samples from 57 small-scale poultry farms underscores the significant burden of AMR in poultry farming. This high prevalence may suggest a widespread exposure to cephalosporins, possibly due to the indiscriminate use of antibiotics in poultry production in Zambia.^26,45^ Given that poultry serves as a major protein source and is closely linked to human food chains, these results highlight serious public health concerns. Additionally, the high prevalence of ESBL-producing Enterobacterales (80%) highlights a serious public health concern as it limits treatment options.^46^

This study found that cefotaxime-resistant isolates were slightly more frequent in cloacal samples (51.9%) than in meat samples (48.1%), with *E. coli* being the predominant bacterial species (77.8%). The significantly higher presence of *E. coli* in cloacal samples than in meat samples (*P* = 0.0013) suggests that resistance primarily originates within the chicken gut microbiota before transmission to meat during processing. Furthermore, presence of clinically important pathogens on market-ready chicken meat samples suggests compromised food safety measures along the food chain posing a serious risk to public health. *K. pneumoniae*, *A. baumannii* and *P. aeruginosa* are known for their role in nosocomial infections and MDR,^47,48^ thus their presence in chicken meat further raises serious concerns about zoonotic transmission and their contribution to the overall AMR crisis in human medicine.

The AST results revealed alarming levels of resistance to ampicillin, tetracycline and co-trimoxazole that could be attributed to excessive usage of penicillins, tetracyclines and sulphonamides in Zambia. This conjecture is supported by a survey that found that tetracyclines, sulfonamides and penicillins were the most sold antibiotics to poultry farmers in Zambia.^5^ Furthermore, despite some antibiotic classes being used less, resistance can be co-selected if the involved AMR genes are present on the same plasmid.^21^ This could explain why resistance was observed across all eight antimicrobials studied here. Currently, carbapenem resistance is rare in Zambian human hospitals, therefore, detecting imipenem resistance in this study raises concern as clonal transmission of MDR *E. coli* between humans and poultry has been reported in Zambia.^49^ While the isolates reported here are probably non-pathogenic, resistance can be transmitted to pathogenic strains via mobile genetic elements such as plasmids, insertion sequences, transposons and translocatable units.^50^

All 3GC-resistant Enterobacterales are placed in the critical tier of the updated WHO Bacterial Priority Pathogens List (BPPL).^51^ In this study, three species (*E. coli*, *K. pneumoniae* and *Enterobacter* spp.) belonging to the Enterobacterales order were identified. In addition, although not on WHO BPPL, cefotaxime-resistant but imipenem-susceptible *Pseudomonas* spp., *A. baumannii* and *C. aquatica* were isolated. *A. baumannii* is mainly associated with hospital acquired infections, especially the carbapenem-resistant isolates.^52^ Recently, Zambia has reported drug-resistant *Acinetobacter* species isolated from the University Teaching Hospital, the most prevalent being *A. baumannii*.^53^ However, *A. baumannii* has recently been isolated from poultry farms globally. Poultry farms, especially those using intensive farming systems, provide an environment where *A. baumannii* can persist and spread.^54–56^ Therefore, our findings emphasize the risk posed by poultry as a reservoir for resistant pathogens.

On the other hand, *C. aquatica* is frequently isolated from wastewater and soil,^57^ thus, the presence of *C. aquatica* on chicken meat is highly suggestive of environmental contamination during processing. This contamination of chicken meat may occur through multiple ways during processing, production and handling.^15^ Additionally, contaminated feed or water can also introduce bacteria that can then colonize the intestines and eventually the meat during processing.^15^

A dendrogram based on AST results and *bla* genes was constructed to cluster isolates based on their AMR profiles. The primary goal was to group isolates based on their phenotypic resistance characteristics, rather than by their taxonomic and evolutionary links. The clustering of *Pseudomonas* spp., *P. aeruginosa* and *A. baumannii* in CB4 was characterized by absence of *bla* genes, which could be due to presence of other *bla* genes or those encoding AmpC β-lactamases.

All isolates in this study were screened for four *bla* genes with *bla*_CTX-M_ and *bla*_TEM_ being the most detected, while *bla*_OXA-1-like_ was less common, and *bla*_SHV_ was only detected in one isolate. Meanwhile, most of the *E. coli* isolates were positive for at least one *bla* gene, with almost half harbouring the *bla*_CTX-M_ and *bla*_TEM_ in co-existence. This observation could be due to these genes co-existing on the same plasmid and can be easily transferred between and within bacterial species.^58,59^ The common occurrence of *bla*_CTX-M_ and *bla*_TEM_ genes in 3GC-resistant *E. coli* has been previously reported in Zambia.^5^ While five groups of the *bla*_CTX-M_ gene exist (i.e. groups 1, 2, 8, 9 and 25), whole-genome-based studies in Zambia have only found those belonging to groups 1 and 2.^49^ Therefore, this study targeted these alleles using primers CTX-MA1/CTX-MA2, specific for *bla*_CTX-M_ groups 1, 2 and 9.

Therefore, the absence of *bla*_CTX-M_ genes in some cefotaxime-resistant isolates requires further probing. For instance, the cefotaxime resistance observed in *A. baumannii*, *Pseudomonas* spp., *P. aeruginosa* and *Enterobacter* spp. could be attributed to intrinsic AmpC β-lactamase activity, which is frequently combined with the activity of efflux pumps and together significantly contribute to resistance against this cephalosporin antibiotic.^60–62^ The observed cefotaxime resistance in these strains could also be due to rare ESBLs or point mutations affecting porins and penicillin-binding proteins. Furthermore, we used universal primers to detect *bla*_TEM_ and *bla*_SHV_; thus, a positive result does not guarantee cefotaxime resistance as some alleles (e.g. *bla*_TEM-1_, *bla*_TEM-2_ and *bla*_SHV-1_) encode narrow-spectrum β-lactamases, which cannot hydrolyse 3GCs. Finally, we used primers targeting *bla*_OXA-1-like_ alleles, whose product inactivates amino and ureidopenicillins and early generation cephalosporins, but not 3GCs.

Only four AMR genes were assessed; therefore, whole genome sequencing (WGS) is needed to identify other AMR genes (such as AmpC-encoding *bla* genes), mobile genetic elements and other resistance mechanisms. WGS would also characterize the strains further regarding OH serotypes, sequence types and plasmid replicons. Additionally, this study did not include *E. coli* phylogenetics, we hope future studies include molecular typing of poultry isolates to characterize the prevailing genotypes in Zambia. This study was limited to two districts within the same province, located ∼63 km apart, a region with high-risk for AMR.^7,63,64^ The findings of this study may not accurately represent AMR trends in Zambia but may be extrapolated to other provinces with a high density of small-scale poultry farms especially in Lusaka and Central Provinces.^4^ Nonetheless, a larger scale study is needed to capture a representative AMR situation in poultry in Zambia.

### Conclusion

This study found cefotaxime-resistant and β-lactamase-producing Enterobacterales in poultry from the Copperbelt province of Zambia. The isolated cefotaxime-resistant strains belonging to three species of Enterobacterales were differently distributed between cloacal and meat swabs. AST patterns revealed high MDR prevalence, contributing more to resistance to the commonly sold antibiotic classes in Zambia (i.e. penicillins, tetracyclines and sulphonamides). Further studies are needed to identify contamination sources and ensure the food safety of poultry meat. In addition, carbapenem resistance should be closely monitored by routine ASTs and WGS.

## Supplementary Material

dlaf125_Supplementary_Data

## Supplementary data

Tables S1 to S3 are available as Supplementary data at *JAC-AMR* Online.
