# Supplementary material for: Occurrence of cefotaxime-resistant and beta-lactamase-producing Enterobacterales in poultry from small-scale farms in the Copperbelt province of Zambia
Source: JAC Antimicrob Resist. 2025 Jul 22;7(4):dlaf125. doi: 10.1093/jacamr/dlaf125 (PMC12280279; doi:10.1093/jacamr/dlaf125)
Supplement: dlaf125_Supplementary_Data [file dlaf125_supplementary_data.docx]

**SUPPLEMENTARY TABLES**

**Table S1:** Species according to district, sample source and *bla* genes

| **ID** | **Species** | **CTX-M** | **TEM** | **SHV** | **OXA-1-like** | **Sample source** | **District** |
| --- | --- | --- | --- | --- | --- | --- | --- |
| ND01MA | *E. coli* | Positive | Negative | Negative | Negative | Muscle | Ndola |
| ND01MB | *A. baumannii* | Negative | Negative | Negative | Negative | Muscle | Ndola |
| ND02C | *E. coli* | Positive | Positive | Negative | Negative | Cloaca | Ndola |
| ND02MA | *E. coli* | Positive | Negative | Negative | Negative | Muscle | Ndola |
| ND02MB | *E. coli* | Positive | Negative | Negative | Negative | Muscle | Ndola |
| ND03C | *E. coli* | Positive | Positive | Negative | Negative | Cloaca | Ndola |
| ND03M | *A. baumannii* | Positive | Positive | Negative | Negative | Muscle | Ndola |
| ND04C | *E. coli* | Positive | Positive | Negative | Negative | Cloaca | Ndola |
| ND04M | *E. coli* | Positive | Positive | Negative | Negative | Muscle | Ndola |
| ND05C | *E. coli* | Positive | Positive | Negative | Negative | Cloaca | Ndola |
| ND05M | *E. coli* | Positive | Negative | Negative | Negative | Muscle | Ndola |
| ND06C | *E. coli* | Positive | Positive | Negative | Negative | Cloaca | Ndola |
| ND06M | *E. coli* | Positive | Positive | Negative | Negative | Muscle | Ndola |
| ND07C | *E. coli* | Negative | Positive | Negative | Negative | Cloaca | Ndola |
| ND07MA | *K. pneumoniae* | Positive | Positive | Negative | Negative | Muscle | Ndola |
| ND07MB | *A. baumannii* | Negative | Negative | Negative | Negative | Muscle | Ndola |
| ND08C | *E. coli* | Positive | Negative | Negative | Negative | Cloaca | Ndola |
| ND08MA | *E. coli* | Positive | Negative | Negative | Negative | Muscle | Ndola |
| ND08MB | *P. aeruginosa* | Negative | Negative | Negative | Negative | Muscle | Ndola |
| ND09C | *E. coli* | Positive | Negative | Negative | Negative | Cloaca | Ndola |
| ND09MA | *E. coli* | Positive | Negative | Negative | Negative | Muscle | Ndola |
| ND09MB | *E. coli* | Negative | Negative | Negative | Negative | Muscle | Ndola |
| ND10C | *E. coli* | Negative | Positive | Negative | Negative | Cloaca | Ndola |
| ND10M | *E. coli* | Negative | Positive | Negative | Negative | Muscle | Ndola |
| KT11C | *E. coli* | Negative | Positive | Negative | Negative | Cloaca | Kitwe |
| KT11M | *E. coli* | Positive | Positive | Negative | Negative | Muscle | Kitwe |
| KT12C | *E. coli* | Positive | Positive | Negative | Positive | Cloaca | Kitwe |
| KT12MA | *E. coli* | Negative | Negative | Negative | Negative | Muscle | Kitwe |
| KT12MB | *A. baumannii* | Negative | Negative | Negative | Negative | Muscle | Kitwe |
| KT13C | *E. coli* | Negative | Positive | Negative | Negative | Cloaca | Kitwe |
| KT13MA | *K. pneumoniae* | Positive | Positive | Negative | Positive | Muscle | Kitwe |
| KT13MB | *C. aquatica* | Negative | Negative | Negative | Positive | Muscle | Kitwe |
| KT14C | *E. coli* | Positive | Positive | Negative | Positive | Cloaca | Kitwe |
| KT15C | *E. coli* | Negative | Positive | Negative | Negative | Cloaca | Kitwe |
| KT15MA | *K. pneumoniae* | Positive | Negative | Negative | Positive | Muscle | Kitwe |
| KT16C | *E. coli* | Positive | Negative | Negative | Negative | Cloaca | Kitwe |
| KT16MA | *K. pneumoniae* | Positive | Negative | Negative | Negative | Muscle | Kitwe |
| KT16MB | *P. aeruginosa* | Negative | Negative | Negative | Negative | Muscle | Kitwe |
| KT17C | *E. coli* | Negative | Negative | Negative | Negative | Cloaca | Kitwe |
| KT17MA | *K. pneumoniae* | Positive | Negative | Positive | Positive | Muscle | Kitwe |
| KT17MB | *Enterobacter spp.* | Negative | Negative | Negative | Negative | Muscle | Kitwe |
| ND22C | *E. coli* | Positive | Positive | Negative | Positive | Cloaca | Ndola |
| ND24C | *E. coli* | Positive | Positive | Negative | Negative | Cloaca | Ndola |
| ND25C | *E. coli* | Positive | Positive | Negative | Negative | Cloaca | Ndola |
| ND26CA | *E. coli* | Positive | Positive | Negative | Negative | Cloaca | Ndola |
| ND26CB | *E. coli* | Positive | Negative | Negative | Negative | Cloaca | Ndola |
| ND27C | *E. coli* | Negative | Negative | Negative | Negative | Cloaca | Ndola |
| KT29CA | *E. coli* | Positive | Positive | Negative | Negative | Cloaca | Kitwe |
| KT29CB | *E. coli* | Positive | Positive | Negative | Negative | Cloaca | Kitwe |
| KT29M | *E. coli* | Positive | Positive | Negative | Negative | Muscle | Kitwe |
| KT30C | *E. coli* | Positive | Positive | Negative | Negative | Cloaca | Kitwe |
| KT33CA | *E. coli* | Positive | Positive | Negative | Negative | Cloaca | Kitwe |
| KT33CB | *E. coli* | Positive | Positive | Negative | Positive | Cloaca | Kitwe |
| KT33M | *E. coli* | Negative | Negative | Negative | Negative | Muscle | Kitwe |
| KT34C | *E. coli* | Positive | Positive | Negative | Negative | Cloaca | Kitwe |
| ND39CA | *E. coli* | Positive | Positive | Negative | Positive | Cloaca | Ndola |
| ND39CB | *E. coli* | Positive | Positive | Negative | Positive | Cloaca | Ndola |
| ND40C | *E. coli* | Positive | Negative | Negative | Negative | Cloaca | Ndola |
| ND40M | *E. coli* | Negative | Negative | Negative | Negative | Muscle | Ndola |
| ND45C | *E. coli* | Positive | Positive | Negative | Negative | Cloaca | Ndola |
| ND46M | *E. coli* | Positive | Positive | Negative | Negative | Muscle | Ndola |
| ND48C | *E. coli* | Positive | Positive | Negative | Negative | Cloaca | Ndola |
| KT55C | *E. coli* | Positive | Positive | Negative | Negative | Cloaca | Kitwe |
| KT56C | *E. coli* | Positive | Positive | Negative | Negative | Cloaca | Kitwe |
| KT56M | *Pseudomonas spp.* | Negative | Negative | Negative | Negative | Muscle | Kitwe |
| KT57C | *E. coli* | Negative | Negative | Negative | Negative | Cloaca | Kitwe |
| KT62M | *Pseudomonas spp.* | Negative | Negative | Negative | Negative | Muscle | Kitwe |
| ND18C | *E. coli* | Positive | Negative | Negative | Negative | Cloaca | Ndola |
| ND19MA | *E. coli* | Negative | Negative | Negative | Negative | Muscle | Ndola |
| ND19MB | *Pseudomonas spp.* | Negative | Negative | Negative | Negative | Muscle | Ndola |
| ND23CA | *E. coli* | Negative | Negative | Negative | Negative | Cloaca | Ndola |
| ND23CB | *E. coli* | Negative | Negative | Negative | Negative | Cloaca | Ndola |
| ND26M | *E. coli* | Negative | Negative | Negative | Negative | Muscle | Ndola |
| ND28CA | *E. coli* | Negative | Negative | Negative | Negative | Cloaca | Ndola |
| ND28CB | *E. coli* | Negative | Negative | Negative | Negative | Cloaca | Ndola |
| ND28M | *E. coli* | Negative | Negative | Negative | Negative | Muscle | Ndola |
| KT29MA | *E. coli* | Negative | Negative | Negative | Negative | Muscle | Kitwe |
| KT29MB | *E. coli* | Negative | Negative | Negative | Negative | Muscle | Kitwe |
| KT30M | *Pseudomonas spp.* | Negative | Negative | Negative | Negative | Muscle | Kitwe |
| KT31C | *E. coli* | Positive | Negative | Negative | Negative | Cloaca | Kitwe |
| KT34M | *Pseudomonas spp.* | Negative | Negative | Negative | Negative | Muscle | Kitwe |

**Table S2: *E. coli* prevalence per number of chickens sampled on a farm**

| **No. of chickens sampled per chicken population** | **A^x^** | **B^y^** | **C^z^** | **Totals** |
| --- | --- | --- | --- | --- |
| **No. of farms sampled** | 28 | 20 | 9 | **57** |
| **Overall no. of chickens on farms per group (i.e A,B,C)** | 28 | 40 | 27 | **95** |
| **No. of pooled samples with at least one *E. coli* isolate-overall** | 16 | 15 | 10 | **41** |
| **No. of pooled samples with at least one *E. coli* isolate-cloaca** | 14 | 15 | 9 | **38** |
| **No. of pooled samples with at least one *E. coli* isolate-meat** | 7 | 8 | 4 | **19** |
| ***p*-value group A vs B** | 0.18 | | | |
| ***p*-value group A vs C** | 0.22 | | | |
| ***p*-value group B vs C** | 1 | | | |

X=1 chicken sampled per 100 birds per farm, Y=2 chickens sampled per 200 birds per farm,

Z=3 chcickens sampled per 300 birds or more per farm.

**Table S3**: Combination disk testing with cefpodoxime (CPD) and cefpodoxime/ clavulanic acid (CD01)

| ID | CPD | CD01 | Zone difference | ESBL production | Species | CTX-M | TEM | SHV | OXA-1-like |
| --- | --- | --- | --- | --- | --- | --- | --- | --- | --- |
| ND01MA | 8 | 27 | 19 | Positive | *E. coli* | Positive | Negative | Negative | Negative |
| ND02C | 6 | 26 | 20 | Positive | *E. coli* | Positive | Positive | Negative | Negative |
| ND02MB | 6 | 25 | 19 | Positive | *E. coli* | Positive | Negative | Negative | Negative |
| ND03C | 6 | 25 | 19 | Positive | *E. coli* | Positive | Positive | Negative | Negative |
| ND04C | 6 | 26 | 20 | Positive | *E. coli* | Positive | Positive | Negative | Negative |
| ND04M | 6 | 11 | 5 | Positive | *E. coli* | Positive | Positive | Negative | Negative |
| ND05C | 8 | 25 | 17 | Positive | *E. coli* | Positive | Positive | Negative | Negative |
| ND05M | 6 | 23 | 17 | Positive | *E. coli* | Positive | Negative | Negative | Negative |
| ND06C | 6 | 22 | 16 | Positive | *E. coli* | Positive | Positive | Negative | Negative |
| ND06M | 6 | 25 | 19 | Positive | *E. coli* | Positive | Positive | Negative | Negative |
| ND07C | 6 | 23 | 17 | Positive | *E. coli* | Negative | Positive | Negative | Negative |
| ND07MA | 6 | 21 | 15 | Positive | *K. pneumoniae* | Positive | Positive | Negative | Negative |
| ND08C | 10 | 23 | 13 | Positive | *E. coli* | Positive | Negative | Negative | Negative |
| ND08MA | 6 | 23 | 17 | Positive | *E. coli* | Positive | Negative | Negative | Negative |
| ND09C | 6 | 28 | 22 | Positive | *E. coli* | Positive | Negative | Negative | Negative |
| ND09MA | 6 | 26 | 20 | Positive | *E. coli* | Positive | Negative | Negative | Negative |
| KT11M | 6 | 27 | 21 | Positive | *E. coli* | Positive | Positive | Negative | Negative |
| KT12C | 6 | 25 | 19 | Positive | *E. coli* | Positive | Positive | Negative | Positive |
| KT13MA | 6 | 25 | 19 | Positive | *K. pneumoniae* | Positive | Positive | Negative | Positive |
| KT15C | 6 | 22 | 16 | Positive | *E. coli* | Negative | Positive | Negative | Negative |
| KT15MA | 6 | 20 | 14 | Positive | *K. pneumoniae* | Positive | Negative | Negative | Positive |
| KT16C | 6 | 24 | 18 | Positive | *E. coli* | Positive | Negative | Negative | Negative |
| KT16MA | 6 | 25 | 19 | Positive | *K. pneumoniae* | Positive | Negative | Negative | Negative |
| KT17MA | 6 | 27 | 21 | Positive | *K. pneumoniae* | Positive | Negative | Positive | Positive |
| ND25C | 6 | 24 | 18 | Positive | *E. coli* | Positive | Positive | Negative | Negative |
| ND26CA | 6 | 25 | 19 | Positive | *E. coli* | Positive | Positive | Negative | Negative |
| ND26CB | 10 | 28 | 18 | Positive | *E. coli* | Positive | Negative | Negative | Negative |
| KT29CA | 6 | 23 | 17 | Positive | *E. coli* | Positive | Positive | Negative | Negative |
| KT29M | 6 | 25 | 19 | Positive | *E. coli* | Positive | Positive | Negative | Negative |
| KT33CA | 6 | 24 | 18 | Positive | *E. coli* | Positive | Positive | Negative | Negative |
| KT33CB | 6 | 23 | 17 | Positive | *E. coli* | Positive | Positive | Negative | Positive |
| ND39CB | 10 | 24 | 14 | Positive | *E. coli* | Positive | Positive | Negative | Positive |
| ND26M | 16 | 19 | 3 | Negative | *E. coli* | Negative | Negative | Negative | Negative |
| ND10C | 6 | 6 | 0 | Negative | *E. coli* | Negative | Positive | Negative | Negative |
| ND10M | 6 | 6 | 0 | Negative | *E. coli* | Negative | Positive | Negative | Negative |
| KT11C | 6 | 6 | 6 | Negative | *E. coli* | Negative | Positive | Negative | Negative |
| ND03M | 21 | 24 | 3 | Negative | *A. baumannii* | Positive | Positive | Negative | Negative |
| KT13C | 6 | 6 | 0 | Negative | *E. coli* | Negative | Positive | Negative | Negative |
| KT17C | 6 | 6 | 0 | Negative | *E. coli* | Negative | Negative | Negative | Negative |
| ND22C | 9 | 13 | 4 | Negative | *E. coli* | Positive | Positive | Negative | Positive |
